# Calcium entry mediates hyperglycemia-induced apoptosis through Ca^2+^/calmodulin-dependent kinase ll in retinal capillary endothelial cells

**Published:** 2012-09-20

**Authors:** Jun Li, Peipei Wang, Songping Yu, Zhi Zheng, Xun Xu

**Affiliations:** 1 Department of Ophthalmology, Shanghai First People’s Hospital Affiliated Shanghai Jiao Tong University, Shanghai, PR China; 2 Department of Ophthalmology, Lishui City Center Hospital, Zhejiang Province, PR China

## Abstract

**Purpose:**

Hyperglycemia-induced vascular cell apoptosis is a seminal early event in diabetic retinopathy. Prolonged hyperglycemia is known to increase intracellular cytosolic free calcium ([Ca^2+^]i) in retinal vascular endothelial cells (RECs), suggesting that [Ca^2+^]i is a critical trigger for microvascular degeneration. This study aims to elucidate Ca^2+^-dependent signaling mechanisms that mediate hyperglycemia-induced apoptosis in RECs.

**Methods:**

A cultured macaque choroid-retinal endothelial cell line (RF/6A) was incubated in normal glucose (NG), NG plus the Ca^2+^ entry blocker 2-aminoethoxydiphenyl borate (2-APB), high glucose (HG), or HG plus either 2-APB, the c-jun N-terminal kinase (JNK) inhibitor SP600125, or the calcium/calmodulin-dependent protein kinase II (CaMKII) inhibitor KN93. Changes in [Ca^2+^]i evoked by adenosine 5′-triphosphate (ATP) were measured in fluo-3/AM-loaded RF/6A cells by confocal microscopy. The mitochondrial membrane potential (ΔΨm) and apoptosis were assessed by flow cytometry. Expression levels of CaMKII, phosphorylated CaMKII (p-CaMKII), c-Jun N-terminal kinase (JNK), phosphorylated JNK (p-JNK), the death receptor (Fas), and cytochrome c were detected by western blotting analysis.

**Results:**

Prolonged exposure to HG (96 h) potentiated ATP-evoked Ca^2+^ entry as well as CaMKII phosphorylation and RF/6A cell apoptosis. Enhanced apoptosis was blocked by 2-APB and KN93. Furthermore, HG increased JNK phosphorylation and Fas expression, and both responses were partially blocked by 2-APB and KN93, while the JNK inhibitor SP600125 partially reduced HG-induced Fas expression. In addition, HG depolarized the ΔΨm and triggered the release of mitochondrial cytochrome c. These early signs of mitochondria-dependent apoptosis were partially reversed by 2-APB and KN93.

**Conclusions:**

HG-induced apoptosis in RF/6A cells depends on Ca^2+^ entry and CaMKII activation, leading to the activation of both Fas-dependent and mitochondria-dependent apoptosis pathways. The CaMKII−JNK−Fas pathway is involved in HG-evoked apoptosis of RECs.

## Introduction

Diabetic retinopathy (DR) is the leading cause of new-onset blindness among the working-age population in developed countries [1]. DR is characterized by early loss of vascular endothelial cells, leading to retinal microvascular dysfunction. High ambient glucose has been shown to promote apoptosis in cultured retinal endothelial cells in vitro [2], and significant apoptosis of retinal endothelial cells has also been detected in a rat model of DR [3]. Therefore, a major focus in the development of new DR treatments is on the inhibition of hyperglycemia-induced retinal capillary endothelial cell apoptosis.

Ca^2+^ is a major trigger of endothelial cell apoptosis [4]. Indeed, hyperglycemia-induced apoptosis in human umbilical vein endothelial cells (HUVECs) requires cytoplasmic Ca^2+^ influx through store-operated channels (SOC) [5]. Recent studies have identified members of the canonical transient receptor potential (TRPC) subfamily of cation channels as the most likely mediators of hyperglycemia-induced Ca^2+^ influx in endothelial cells [6]. However, the downstream signaling pathways through which Ca^2+^ triggers apoptosis under hyperglycemia have not been fully elucidated.

The Ca^2+^/calmodulin-dependent protein kinase II (CaMKII), a multifunctional enzyme that catalyzes the phosphorylation of a myriad of eukaryotic proteins, is activated upon both sustained intracellular cytosolic free calcium ([Ca^2+^]i) increases and [Ca^2+^]i oscillations, and serves to translate these [Ca^2+^]i signals into cellular responses [7]. Activated CaMKII is an important mediator of retinal cell apoptosis in diabetes [8,9]. Recently, it was demonstrated that CaMKII is essential for both endoplasmic reticulum (ER) stress-induced apoptosis through the death receptor Fas, and for mitochondria-dependent apoptosis. Mitochondrial apoptosis involves the activation of the c-jun N-terminal kinase (JNK), which leads to outer mitochondrial membrane permeabilization, cytochrome c release, and the activation of the caspase-dependent apoptotic cascade [10]. ER stress is a central feature of type 2 diabetes and its chronic complications, such as DR [11,12].

Based on these results, we proposed that CaMKII is a central mediator of hyperglycemia-induced apoptosis in retinal capillary endothelial cells. This study aimed to test this possibility, and we measured ATP-stimulated Ca^2+^ release in a cultured macaque choroid-retinal endothelial cell line (RF/6A) in normal and high glucose concentrations, and investigated the downstream signaling mechanisms involved in Ca^2+^ entry and CaMKII activation in RF/6A cells stimulated with high glucose (HG). We demonstrated that CaMKII contributes to hyperglycemia-induced RF/6A cells apoptosis by activating both Fas-dependent and mitochondrial apoptosis pathways, suggesting that CaMKII is an important therapeutic target for DR.

## Methods

### Cell culture and materials

A macaque choroid-retinal endothelial cell line (RF/6A) was obtained from the cell bank of the Chinese Academy of Science (CAS, Shanghai, China) and cultured as described previously [13]. Briefly, RF/6A cells were cultured in Dulbecco’s modified Eagle’s medium (DMEM; Invitrogen, Carlsbad, CA) supplemented with 10% fetal bovine serum (FBS; ScienceCell, San Diego, CA), 100 U/ml penicillin (Invitrogen), 100 mg/ml streptomycin (Invitrogen) at 37 °C in a humidified atmosphere containing 5% CO_2_ and 95% humidified air. Cultured RF/6A cells at passages 3 or 4 were used in the experiments that follow. Confluent RF/6A cells were maintained in DMEM and supplemented with 0.4% BSA. The cells were incubated for 96 h in normal D-glucose (5.5 mM; NG), NG plus 24.5 mM D-mannitol (NG+D-mannitol), NG plus 100 μM 2-APB (a Ca^2+^ entry blocker), high D-glucose (HG, 30 mM), or HG in the presence of 100 μM 2-APB, 10 μM SP600125 (a JNK inhibitor), or 10 μM KN93 (a CaMKII inhibitor) as indicated. All chemicals were of reagent grade and purchased from Sigma Chemicals (St. Louis, MO) unless stated otherwise.

### Determination of [Ca^2+^]i

The RF/6A cells were loaded with 5 μM fluo-3 AM (Invitrogen) for 30 min at 37 °C. After they were rinsed, the cells were viewed using a Zeiss confocal microscope (400× oil immersion objective; Leica Microsystems, Heidelberg, Germany). Furo-3 fluorescence was produced by excitation from a 75-W xenon arc lamp with appropriate filter sets (excitation 488 nm; emission 510/530 nm; Sutter Instruments, Novato, CA). After baseline images were acquired, the cells were stimulated with 200 μM ATP (with or without extracellular Ca^2+^). Image acquisition continued for 10 min and the intensities of the intracellular fluorescence were measured by software Image-Pro Plus 5.1. Briefly, regions of interest were defined by drawing an outline around each cell body, and the mean fluorescence was extracted across the time-lapse sequence of images to obtain fluorescence versus time plots for each cell. Background fluorescence was obtained from a region with no cells for every field examined and subtracted from the mean fluorescence. The mean fluorescence was also corrected for the mean baseline fluorescence determined before the stimulation of the cells. For each treatment condition, 30–35 cells within a single field of view were analyzed.

### Measurement of apoptosis

Apoptosis was assessed by an Annexin V-FITC/propidium iodide (PI) dual staining kit according to the manufacturer’s instructions (Bender Med Systems, Vienna, Austria). Briefly, RF/6A cells were harvested after 96 h exposure to NG or HG (with or without pharmacological inhibitors), washed in cold phosphate-buffered saline (PBS; 130 mM NaCl, 2.5 mM KCl, 8 mM Na_2_HPO_4_, and 1.5 mM KH_2_PO_4_, pH 7.4), and resuspended at 1×10^6^ cells/ml in a binding buffer containing 0.01 M HEPES pH 7.4; 0.14 M NaCl; and 2.5 mM CaCl_2_. Annexin V-FITC (5 μl) and PI (10 μl) were added to the cell suspension (100 μl), vortexed, and incubated for 15 min in the dark at room temperature. Stained cells from each treatment group were analyzed by flow cytometry (FACS Caliber; Becton Dickinson, Heidelberg, Germany). For each sample, data from 10,000 cells was recorded in list mode on logarithmic scales. Analysis was performed with Cell Quest software (BD Biosciences, San Jose, CA) on cells characterized by their forward/side scatter (FSC/SSC) parameters. Cells analyzed included living cells with normal FSC/SSC parameters and dying cells with altered FSC/SSC. Cell debris characterized by a low FSC/SSC and an Annexin V/PI phenotype was excluded from the analysis.

### Measurement of mitochondrial membrane potential

Changes in the mitochondrial membrane potential (ΔΨm) associated with apoptosis were analyzed with the cationic lipophilic fluorescent probe 5,5′,6,6’-Tetrachloro1,1’,3,3′-tetraethyl-benzimidazolylcarbocyanine iodide (JC-1; Molecular Probes). The ΔΨm measurement was performed using flow cytometry (FACS Caliber, Becton Dickinson, Heidelberg, Germany) as described previously [13]. The fluorescent emission of JC-1 shifted reversibly from red (measured at 590 nm) to green (measured at 530 nm) with decreasing ΔΨm when excited at 488 nm, and the red/green emission ratio provided an estimate of the ΔΨm.

### Western blotting analysis

Approximately 3×10^6^ RF/6A cells were harvested and lysed in a buffer containing 1% Nonidet P40, 10 mM Tris, 200 mM NaCl, 5 mM EDTA, and 10% glycerol plus protease inhibitors (pH 7.0). Lysates from the treated cells were centrifuged at 12,000 × *g* for 20 min at 4 °C, and the cleared supernatants were collected. Protein concentrations in the supernatants were measured using the Bio-Rad DC protein assay (Bio-Rad, Hercules, CA). To analyze cytochrome c in different subcellular fractions, separated mitochondrial and cytosolic fractions were obtained using a cytochrome c releasing apoptosis assay kit.

Fifty micrograms of protein from each sample was subjected to 7.5% sodium dodecyl sulfate-PAGE (SDS–PAGE) using a Bio-Rad miniature slab gel apparatus. Separated proteins were electrophoretically transferred onto nitrocellulose membranes. The membranes were blocked with 5% nonfat dried milk solution and incubated overnight with either partially purified rabbit anti-CaMKII and an phospho-CaMKII polyclonal antibody targeting p-Thr286 (Abcam, Cambridge, MA; 1:500), a rabbit anti-JNK and mouse phospho-JNK polyclonal antibody targeting p-Thr183/p-Tyr185 (Abcam; 1:500), a rabbit anti-Fas polyclonal antibody (Santa Cruz Biotechnology, Santa Cruz, CA; 1:500), or a rabbit anti-cytochrome c polyclonal antibody (Cell Signaling Technology, Danvers, MA; 1:500). Expression of β-actin (monoclonal anti-β-actin; Santa Cruz; 1:1000) was used as an internal control to confirm equivalent protein loading per gel lane. After incubation with a horseradish-peroxidase-conjugated anti-rabbit IgG (Cell Signaling Technology) for 2 h at room temperature, the membranes were evaluated using an enhanced chemiluminescence (ECL) system (Amersham Biosciences, Buckinghamshire, England) according to the manufacturer’s instructions, and the band density was determined by Image J software (NIH, Bethesda, MD). Each experiment was performed at least in triplicate.

### Statistical analysis

Experimental data was expressed as mean±SD. Group means were compared by a one-way ANOVA, followed by Tukey’s post tests, for pair-wise comparisons using a software system (Prism 4.0; GraphPad, San Diego, CA) and a statistical software program (SPSS13.0 for Windows; SPSS, Chicago, IL). A p value less than 0.05 was considered significant.

## Results

### Hyperglycemia increases calcium entry in RF/6A cells

**Figure 1:**
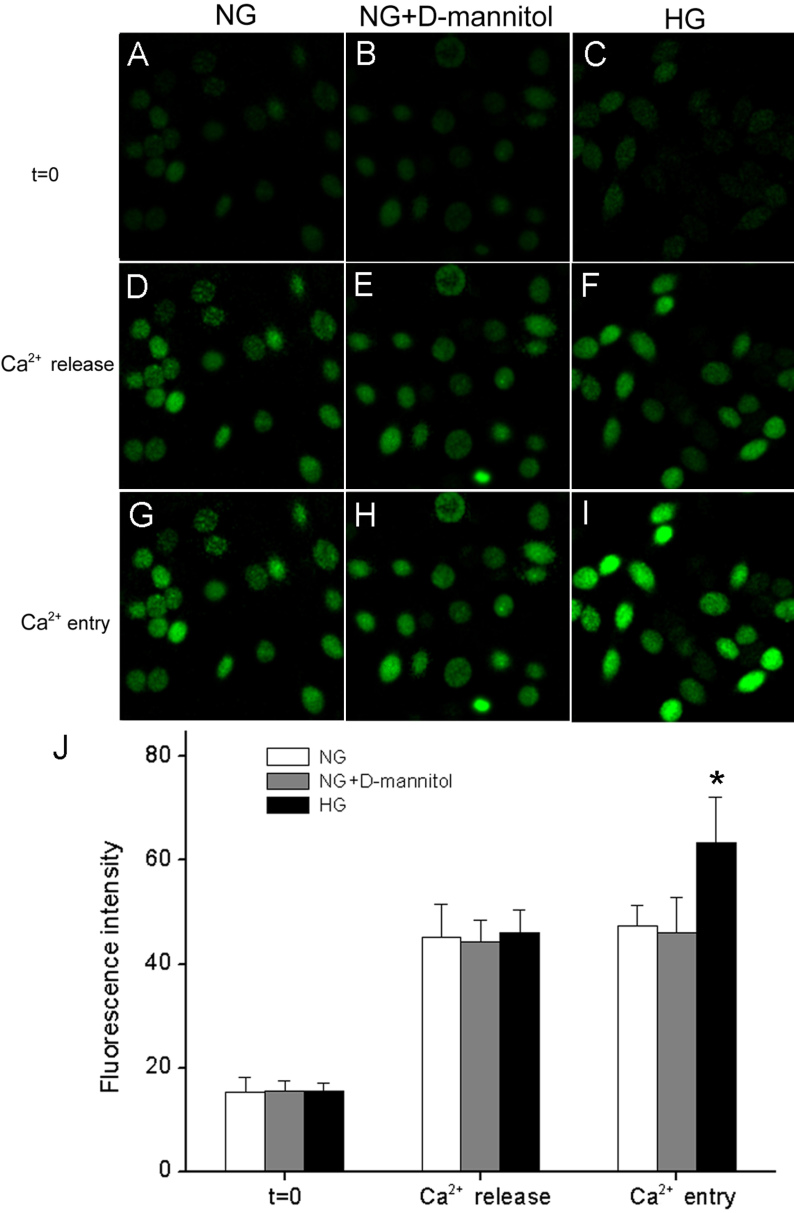
Prolonged hyperglycemia increases ATP-evoked Ca^2+^ influx in RF/6A cells. RF/6A cells were treated for 96 h with 5.5 mM glucose (NG), NG plus 24.5 mM D-mannitol, or 30 mM glucose (HG), and the fluorescence intensity was analyzed as described in Methods. **A**-**C**: Images of fluo-3-loaded cells pre-incubated with NG, NG plus D-mannitol, or HG. **D**-**F**: Images of peak [Ca^2+^]i responses acquired during ATP (200 μM) stimulation in Ca^2+^-free saline (Ca^2+^ release). **G**-**I**: Images of cells after the re-addition of 1.8 mM Ca^2+^ to the extracellular medium (Ca^2+^ entry). **J**: Average baseline [Ca^2+^]i, peak [Ca^2+^]i in Ca^2+^-free saline, and sustained [Ca^2+^]i after the re-addition of extracellular Ca^2+^. Each bar represents the average (SD) of 30–35 cells. * p<0.05 versus NG or NG + D-mannitol.

The resting [Ca^2+^]i was not significantly different between RF/6A cells exposed to 5.5 mM D-glucose (NG) and those exposed to 30 mM D- HG (Figure 1A-C, J). Stimulation of RF/6A cells with ATP caused a biphasic increase in [Ca^2+^]i consisting of an initial transient peak that was also observed in the absence of extracellular Ca^2+^ (Figure 1D-F). The rapid peak was followed by a sustained plateau phase that remained above the original baseline (Figure 1G-I) but required a re-addition of Ca^2+^ to the external medium. Thus, ATP evoked a biphasic [Ca^2+^]i signal mediated by rapid transient release from internal stores and delayed but sustained Ca^2+^ influx. Although the initial peak [Ca^2+^]i under NG was not different from that measured in RF/6A cells preincubated in HG (p>0.05), there was a significant increase (p<0.05) in the sustained phase of Ca^2+^ in cells exposed to HG compared to cells exposed to NG (Figure 1J). To exclude the potential effect of hyperosmolarity on [Ca^2+^]i signals, we used D-mannitol to adjust osmotic pressure. Administration of 24.5 mM D-mannitol in NG media did not significantly affect either the initial peak or the sustained phase of [Ca^2+^]i compared to cells exposed to NG (p>0.05).

### Ca^2+^ entry regulates hyperglycemia-induced apoptosis

**Figure 2:**
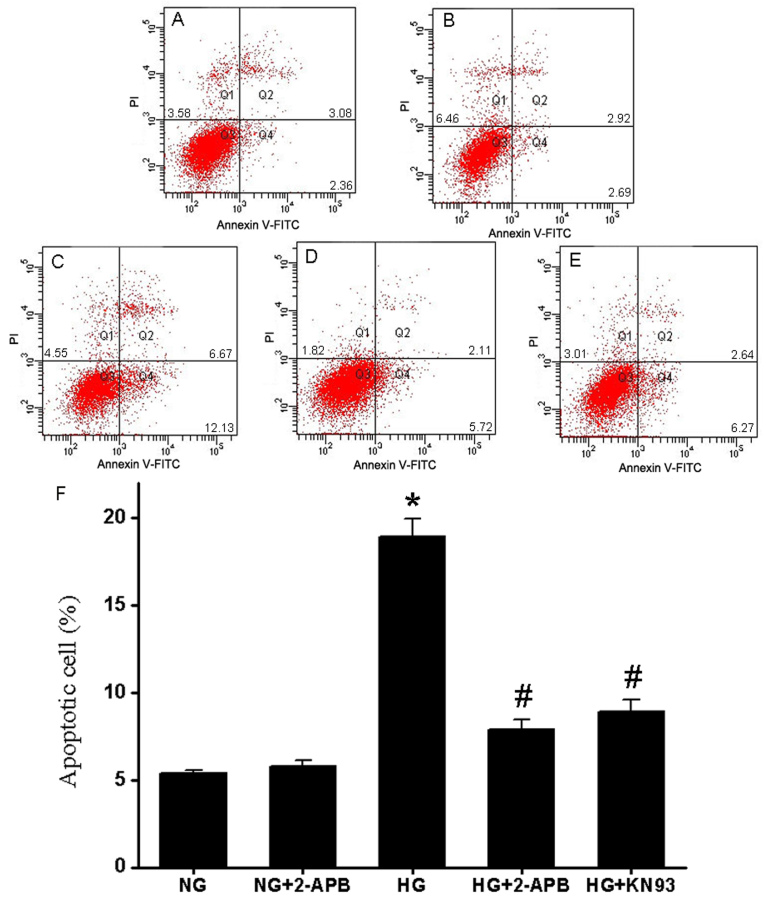
Hyperglycemia induces [Ca^2+^]i-dependent and CaMKll-dependent apoptosis in RF/6A cells. RF/6A cells were treated with 5.5 mM glucose. **A** NG, **B** NG + 2-APB, **C** 30 mM glucose (HG), **D** HG + 2-APB, or **E** HG + KN93 for 96 h, stained by Annexin V/PI and subjected to flow cytometry analysis. Early apoptotic populations were in the lower-right quadrant (Q4, Annexin V-positive) and late apoptotic cells were in the upper-right quadrant (Q2, Annexin V-positive/PI-positive). **F**: Percentages of early and late apoptotic cells under each condition were expressed as the mean±SD of six independent experiments. * p<0.05 versus NG or NG+2-APB; ^#^ p<0.05 versus HG; ^#^ p>0.05 versus NG or NG+2-APB.

To investigate whether HG induced apoptosis under our experimental conditions, treated cells were stained with Annexin V-FITC and PI, and apoptosis was quantified by flow cytometry. Cells were incubated for 96 h in NG (Figure 2A), NG plus 100 μM 2-APB (Figure 2B), HG (Figure 2C), HG plus 100 μM 2-APB (Figure 2D), or HG + 10 μM KN93 (Figure 2E). The number of apoptotic cells under each condition was indicated by two-dimensional dot plots, with dots in the lower-right quadrant (Q4) representing cells in early stage apoptosis (Annexin+/PI-), and those in the upper-right quadrant (Q2) representing cells in late stage apoptosis (Annexin+/PI+). The results showed a significant increase in the number of apoptotic cells in cultures exposed to HG for 96 h (18.80±2.67%) compared to cultures exposed to NG (5.44±1.42%, p<0.05). The increase in the number of apoptotic cells in the hyperglycemic group was reversed in the presence of the Ca^2+^ entry blocker 2-APB, and even reached the level of normal condition (7.83±1.65%, p<0.05 compared with HG, and p>0.05 compared with NG), while 2-APB had no effect on apoptosis in cells incubated in NG (5.61±1.21%, p>0.05 compared with NG). Taken together, this data suggests that Ca^2+^ entry is necessary for hyperglycemia-induced apoptosis in cultured RF/6A cells.

### Hyperglycemia activates CaMKII

**Figure 3:**
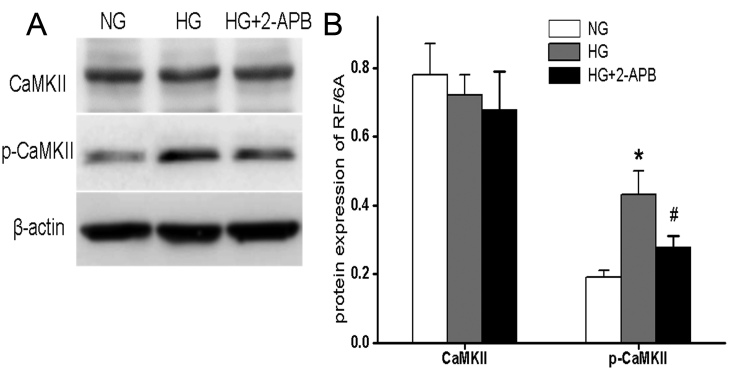
Hyperglycemia promotes CaMKll activation in RF/6A cells. **A**: RF/6A cells were incubated for 96 h in a serum-free medium with 5.5 mM glucose (NG), 30 mM glucose (HG), or HG plus 2-APB, and subjected to western blotting analysis for CaMKll and p-CaMKll protein levels. β-actin served as loading control. **B**: CaMKll and p-CaMKll levels were quantified by densitometry analysis under each treatment condition. Bars represented mean±SD from at least three independent experiments with seven cells per treatment group. * p<0.05 versus NG; ^#^ p<0.05 versus HG; ^#^ p>0.05 versus NG.

The CaMKII is a serine/threonine kinase activated in response to sustained or oscillating increases in [Ca^2+^]i [7]. Thus, we examined the CaMKII protein expression and phospho-activation after 96 h of HG treatment. Western blot analysis showed that the total CaMKII protein levels in RF/6A cells were not significantly changed by HG treatment compared to cells incubated in NG (Figure 3). In contrast, CaMKII kinase activation, as indicated by CaMKII phosphorylation (p-CaMKll expression), was markedly increased in RF/6A cells treated with 30 mM glucose. Notably, a hyperglycemia-induced increase in p-CaMKII expression was blocked by 2-APB, although 2-APB had no effect on total CaMKll expression. The role of CaMKII in HG-induced apoptosis was evaluated by incubating RF/6A cells in HG plus 10 μM KN93 before Annexin V-FITC and PI staining. The results showed that the inhibition of CaMKll activity significantly decreased the number of apoptotic cells after 96 h of treatment with HG compared to HG alone (18.80±2.67% versus 8.91±1.74%, p<0.05; Figure 2), and that the number of apoptotic cells in the HG + CaMKll group was similar to those in the NG condition (8.91±1.74% versus 5.44±1.42%, p>0.05; Figure 2). Taken together, this data suggests that CaMKII plays a critical role in hyperglycemia-induced apoptosis in RF/6A cells.

### Hyperglycemia increases JNK phosphorylation and Fas expression partially through CaMKII

**Figure 4:**
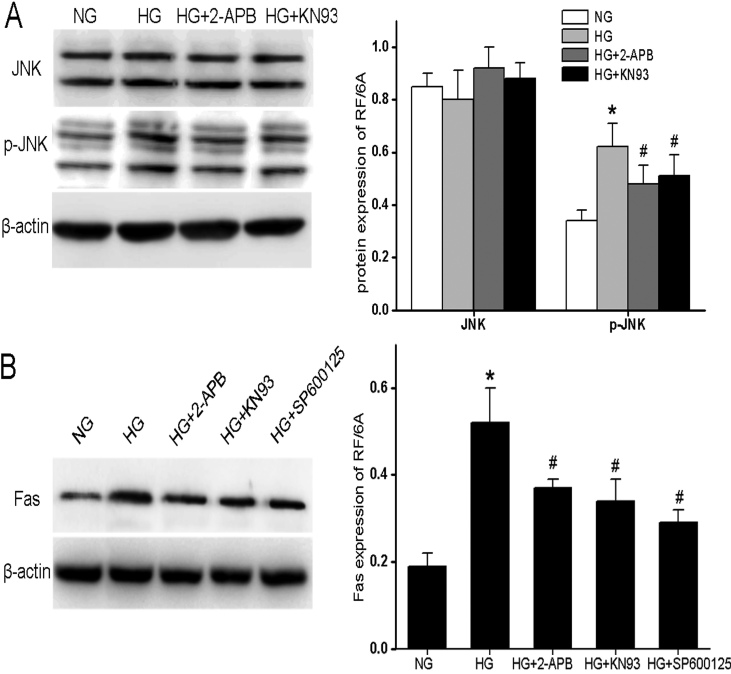
Hyperglycemia induces [Ca^2+^]i-dependent and CaMKll-dependent JNK phosphorylation and Fas protein expression in RF/6A cells. RF/6A cells were incubated for 96 h in serum-free medium containing 5.5 glucose (NG), 30 mM glucose (HG), or HG plus 2-APB, KN93, or SP600125 as indicated, and subjected to western blotting analysis for **A**: JNK, and p-JNK and for **B**: Fas. β-actin served as the loading control. JNK, p-JNK, and Fas levels were quantified by densitometry analysis in each treatment group (right panels). Bars represented mean±SD from at least three independent experiments with seven cells per treatment group. * p<0.05 versus NG; ^#^ p<0.05 versus HG; ^#^ p<0.05 versus NG.

Fas is a death receptor involved in the apoptosis of many cell types, and Fas activation is known to contribute to the development of diabetes and DR [14,15]. Previous reports have suggested the links between CaMKII and JNK [16], and between JNK and Fas induction [17]. We therefore speculated that the activation of a CaMKII−JNK−Fas pathway in RF/6A cells cultured in HG may promote apoptosis. To examine the role of JNK activation (p-JNK) and Fas induction in HG-mediated apoptosis, and the role of CaMKII as an upstream activator, RF/6A cells were incubated for 96 h in serum-free DMEM containing either NG, HG, or HG plus either 100 μM 2-APB or 10 μM KN93. HG induced a significant increase in JNK phosphorylation (Figure 4A) and Fas induction (Figure 4B), and both responses were partially reduced by 2-APB (100 μM) or by KN93 (10 μM). Moreover, treatment of cells with 10 μM SP600125, the JNK inhibitor, also partially reduced HG-induced Fas production, consistent with the activation of a CaMKII−JNK−Fas pathway by HG.

### Release of mitochondrial cytochrome c and loss of mitochondrial membrane potential are CaMKII-dependent in HG-cultured RF/6A cells

**Figure 5:**
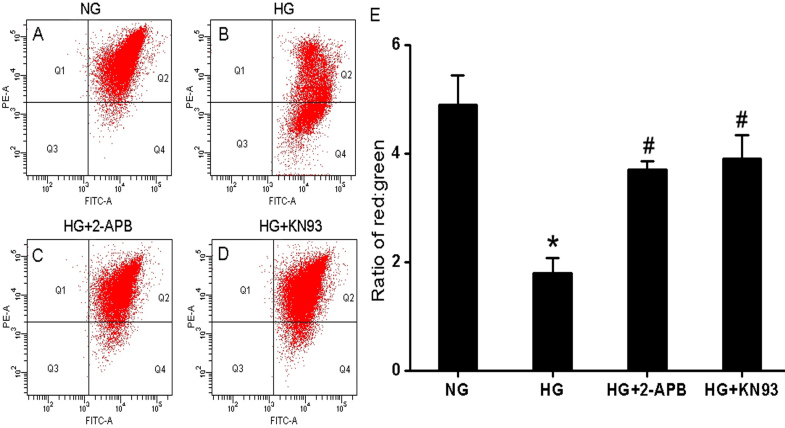
Hyperglycemia evokes [Ca^2+^]i-dependent and CaMKll-dependent mitochondrial membrane depolarization in RF/6A cells. **A**-**D**: Analysis of mitochondrial membrane potential (ΔΨm) in each treatment group. RF/6A cells were treated for 96 h with **A**: 5.5 mM glucose (NG), **B**: 30 mM glucose (HG), **C**: HG plus either 2-APB, or **D**: KN93, and ΔΨm was analyzed by JC-1 staining. Loss of ΔΨm was demonstrated by the change in JC-1 fluorescence from red (JC-1 aggregates) to green (JC-1 monomers). **E**: The bar diagram showed the ratio of JC-1 red fluorescence to green fluorescence under each treatment condition. Data represented mean±SD of three independent experiments. * p<0.05 versus NG; ^#^ p<0.05 versus HG; ^#^ p<0.05 versus NG.

**Figure 6:**
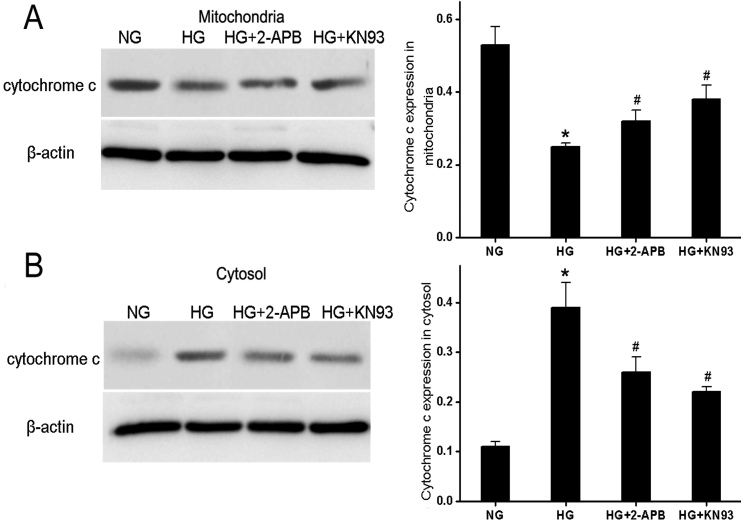
Hyperglycemia causes [Ca^2+^]i-dependent and CaMKll-dependent release of mitochondrial cytochrome c into the cytosol in RF/6A cells. RF/6A cells were treated with 5.5 mM glucose (NG), 30 mM glucose (HG), or HG plus 2-APB or KN93. **A**: Cytochrome c in the mitochondrion was detected by western blotting analysis. **B**: Cytochrom c in the cytoplasm was detected by western blotting analysis. β-actin served as the loading control. The cytochrome c in mitochondrial and cytosolic fractions was quantified by densitometry analysis (right panels) for all treatment groups. Data represented mean±SD from at least three independent experiments with seven cells per group. * p<0.05 versus NG; ^#^ p<0.05 versus HG; ^#^ p<0.05 versus NG.

We have previously shown that the activation of the mitochondrial apoptosis pathway mediates retinal capillary cell death in response to HG in vitro and in diabetic rats in vivo [18]. A drastic increase in the mitochondrial outer member permeability is associated with the loss of the transmembrane potential, ΔΨm, and the release of mitochondrial cytochrome c into the cytosol with ensuing activation of the caspase-dependent apoptosis pathway. To detect changes in ΔΨm indicative of mitochondria-dependent apoptosis, we used the cationic lipophilic fluorochrome JC-1. Incubation of RF/6A cells in HG elicited a decrease in the red/green fluorescence ratio (a green shift) in JC-1 fluorescence emission, indicative of ΔΨm depolarization, while 2-APB or KN93 partially reversed the increase in green fluorescence (Figure 5A-D,E). We then measured cytochrome c release associated with changes in ΔΨm. Incubation of RF/6A cells in HG led to the release of cytochrome c from the mitochondria (Figure 6A) into the cytosol (Figure 6B), and this could be partially suppressed by 2-APB or KN93.

## Discussion

Prolonged hyperglycemia increased ATP-evoked Ca^2+^ entry in retinal capillary endothelial cells. Systematic pharmacological studies indicated that increased Ca^2+^ entry led to phospho-activation of CaMKII, which in turn activated JNK, leading to Fas induction. This signaling pathway activated both Fas-dependent and mitochondria-dependent apoptosis.

It has long been known that [Ca^2+^]i is one of the key upstream signals responsible for the activation of apoptotic pathways [4,5,10,19,20]. Previous studies have shown that hyperglycemia changes extracellular ATP levels in rat retinal cultures and other tissues, and excessive release of extracellular ATP subsequently leads to the changes in intracellular [Ca^2+^]i [21-23]. Therefore, ATP is implicated in the triggering and regulation of the [Ca^2+^]i responses to various stimulation, including hyperglycemia [6,24,25]. Here, using digitized confocal images, we demonstrated that HG exposure increased ATP-induced Ca^2+^ entry but not Ca^2+^ release from intracellular stores, providing evidence for the potential role of ATP-induced Ca^2+^ changes in hyperglycemia, and confirming previous results in other endothelial cells [5,6]. Furthermore, our study demonstrated that the Ca^2+^ entry blocker 2-APB prevented the increase in apoptosis induced by HG, indicating the pivotal role of Ca^2+^ influx in hyperglycemia-induced retinal capillary endothelial cell apoptosis.

CaMKll is a serine/threonine kinase widely distributed in mammalian cells that transduces sustained (graded) Ca^2+^ increases and variable frequency Ca^2+^ spikes into unique cellular responses by phosphorylating distinct subsets of target proteins, including Ca^2+^-dependent cell death effectors [7,26,27]. Moreover, CaMKll has been shown to regulate ion homeostasis, nitric oxide production, and the permeability of endothelial cells [28], and may act as an essential mediator in the development of diabetic vascular dysfunction [29]. In the present study, we demonstrated that selective inhibition of CaMKll activity by KN93 protected RF/6A cells from hyperglycemia-induced apoptosis, consistent with recent studies showing that the activation of CaMKII contributed to the death of other retinal cells in diabetes [8,9].

Our data reveals a possible molecular mechanism through which CaMKII mediates hyperglycemia-induced apoptosis in retinal endothelial cells. The death-receptor and mitochondrial pathways are two major apoptotic pathways in mammalian cells [30]. In the death-receptor pathway, the binding of Fas to its ligand (FasL) activates downstream caspases, such as caspase-8 and caspase-3, that initiate apoptotic death [31]. The mitochondrial pathway is activated by a multitude of extracellular and internal stressors, including DNA damage. The collapse of the mitochondrial membrane potential and cytochrome c release from mitochondria are critical steps in this pathway during cellular Ca^2+^ overload [32]. Here, we demonstrate that the inhibition of CaMKII−JNK signaling partially abrogated hyperglycemia-induced upregulation of Fas. Furthermore, the hyperglycemia-induced decrease in ΔΨm was partially prevented by both the CaMKll inhibitor KN93 and by the Ca^2+^ entry blocker 2-APB. Similarly, both KN93 and 2-APB inhibited CAMKII activation as evidenced by the increased p-CaMKll level, and both partially blocked cytochrome c release from the mitochondria into the cytosol. These results, together with previous findings, establish a critical role for CAMKII activation in hyperglycemia-induced apoptosis in retinal endothelial cells. However, our results do not exclude contributions from other apoptotic signaling pathways. For example, enhanced Ca^2+^ entry concomitant with hyperglycemia may also activate the calcium-dependent phosphatase calcineurin, leading to the dephosphorylation of the pro-apoptotic protein BAD that facilitates apoptosis in other cells [5,33].

In conclusion, the present study demonstrates that Ca^2+^ entry through 2-APB-sensitive channels plays a significant role in hyperglycemia-induced apoptosis in retinal endothelial cells, and this response is at least partially mediated by the activation of CaMKII. In turn, phospho-activated CaMKll activates both Fas-receptor and mitochondrial apoptosis pathways. Inhibition of CaMKII and other strategies targeting specific signaling pathways linking CaMKII to apoptosis may offer therapeutic approaches to a variety of hyperglycemia-induced diseases such as DR.
